# Dynamics of Linker Residues Modulate the Nucleic Acid Binding Properties of the HIV-1 Nucleocapsid Protein Zinc Fingers

**DOI:** 10.1371/journal.pone.0102150

**Published:** 2014-07-16

**Authors:** Loussiné Zargarian, Carine Tisné, Pierre Barraud, Xiaoqian Xu, Nelly Morellet, Brigitte René, Yves Mély, Philippe Fossé, Olivier Mauffret

**Affiliations:** 1 Laboratoire de Biologie et Pharmacologie Appliquée, Ecole Normale Supérieure de Cachan, Centre National de la Recherche Scientifique, Unité Mixte de Recherche 8113, Cachan, France; 2 Laboratoire de Cristallographie et RMN Biologiques, Université Paris Descartes, Centre National de la Recherche Scientifique, Unité Mixte de Recherche 8015, Paris, France; 3 Department of Life Sciences, East China Normal University, Shanghai, People's Republic of China; 4 Centre de Recherches de Gif, Institut de Chimie des Substances Naturelles, Centre National de la Recherche Scientifique, Gif sur Yvette, France; 5 Laboratoire de Biophotonique et Pharmacologie, Centre National de la Recherche Scientifique, Unité Mixte de Recherche 7213, Faculté de Pharmacie, Université de Strasbourg, Illkirch, France; Institut Pasteur, France

## Abstract

The HIV-1 nucleocapsid protein (NC) is a small basic protein containing two zinc fingers (ZF) separated by a short linker. It is involved in several steps of the replication cycle and acts as a nucleic acid chaperone protein in facilitating nucleic acid strand transfers occurring during reverse transcription. Recent analysis of three-dimensional structures of NC-nucleic acids complexes established a new property: the unpaired guanines targeted by NC are more often inserted in the C-terminal zinc finger (ZF2) than in the N-terminal zinc finger (ZF1). Although previous NMR dynamic studies were performed with NC, the dynamic behavior of the linker residues connecting the two ZF domains remains unclear. This prompted us to investigate the dynamic behavior of the linker residues. Here, we collected ^15^N NMR relaxation data and used for the first time data at several fields to probe the protein dynamics. The analysis at two fields allows us to detect a slow motion occurring between the two domains around a hinge located in the linker at the G35 position. However, the amplitude of motion appears limited in our conditions. In addition, we showed that the neighboring linker residues R29, A30, P31, R32, K33 displayed restricted motion and numerous contacts with residues of ZF1. Our results are fully consistent with a model in which the ZF1-linker contacts prevent the ZF1 domain to interact with unpaired guanines, whereas the ZF2 domain is more accessible and competent to interact with unpaired guanines. In contrast, ZF1 with its large hydrophobic plateau is able to destabilize the double-stranded regions adjacent to the guanines bound by ZF2. The linker residues and the internal dynamics of NC regulate therefore the different functions of the two zinc fingers that are required for an optimal chaperone activity.

## Introduction

**Figure 1:**
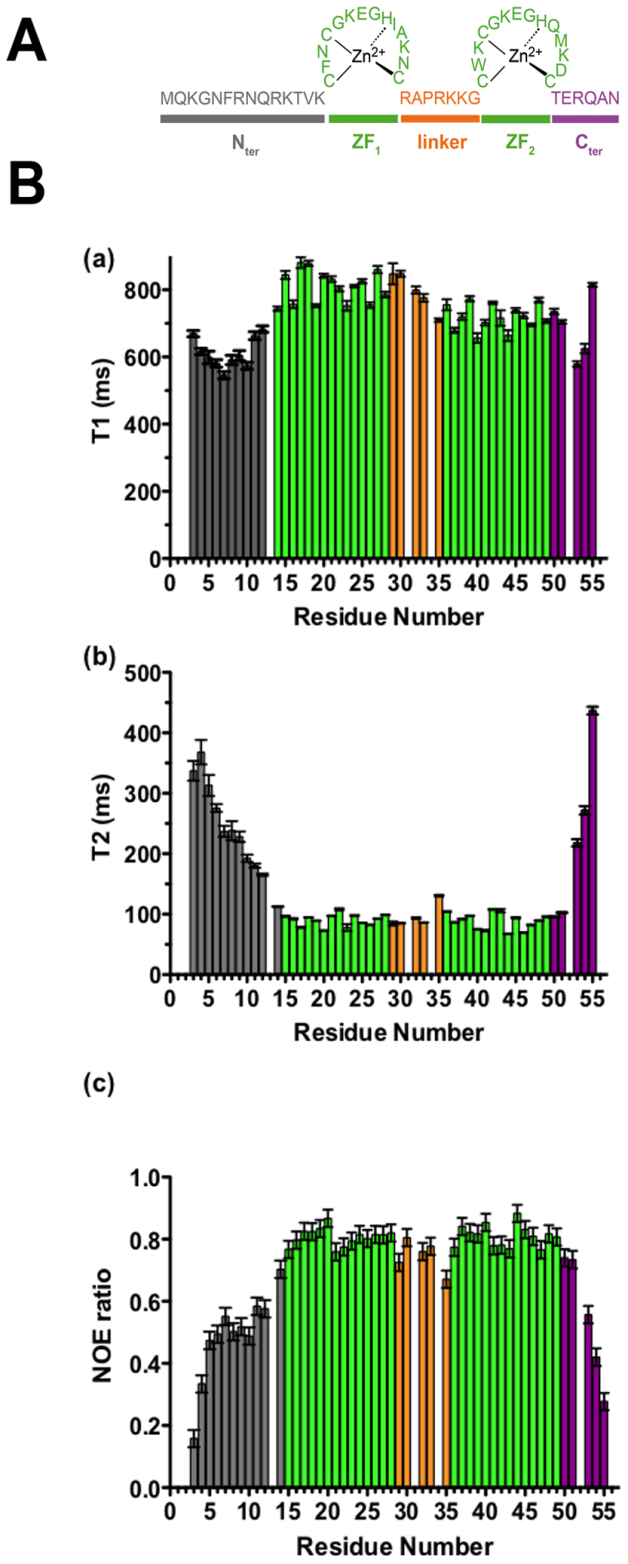
A/Sequence of the HIV-1 nucleocapsid protein (NL4-3). **B/Experimental ^15^N NMR relaxation** **data** (**(a)** Longitudinal (T1), **(b)** transverse (T2) relaxation times and **(c)** heteronuclear nOe) for backbone atoms obtained at 950 MHz for NC at 10°C.

The human immunodeficiency virus type 1 (HIV-1) nucleocapsid protein (NC) is a small nucleic acid binding protein that possesses a N-terminal basic domain and two zinc fingers connected by a short linker (Figure 1A). The NC domain, under its various forms (Gag, NCp15, NCp9, NCp7) plays numerous roles during the replication cycle of the virus [1]–[3]. Among these forms, NCp7 (named NC in this report), via its nucleic acid chaperone activity [1], [4], [5], is thought to facilitate the strand transfer processes occurring during reverse transcription [6]–[9]. Through its chaperone activity, NC rearranges the nucleic acids into the most thermodynamically stable conformations. This activity is mainly related to the ability of the protein: i) to destabilize secondary structures of nucleic acids and ii) to promote annealing/aggregation of nucleic acids. Additionally, the fast kinetics of the binding/unbinding of NC to nucleic acids [10], [11] as well as the freezing of the local mobility of the contacted bases [12], [13] were reported as further key properties of NC chaperone activity. Interestingly, zinc fingers are thought to play a major part in nucleic acid destabilization, fast binding, and dynamic restriction, while the basic N-terminal part is mainly responsible for the nucleic acid aggregation activity [1]–[3], [11], [14]–[19].

NC exhibits a clear preference for single-stranded regions [3], [20]. Moreover, NC exhibits high affinity for oligonucleotides containing unpaired guanines, such as TG, UG, TGG, GXG in internal or apical loops or in single-stranded domains [1], [12], [21]–[25]. The two zinc fingers of NC are involved in these preferences and most of the structural reasons for these preferences have been inferred from the 3D structures of NC complexes with short oligonucleotides [23], [24], [26].

Several studies showed that the two zinc fingers (ZF1 and ZF2) are not equivalent [21], [27]–[30]. Indeed, the NC mutant in which the N-terminal zinc finger (ZF1) has been duplicated (ZF1:ZF1 mutant) is more replication competent than mutants with zinc finger-swap (ZF2:ZF1 mutant) or with duplicated C-terminal finger (ZF2:ZF2 mutant) [29]. Using the same mutants, ZF1 was shown to be more critical than ZF2 for the nucleic acid chaperone activity of NC [28], [29], [31], [32]. Indeed, facilitation by NC of strand transfers as well as annealing of highly structured nucleic acids substrates is observed only when ZF1 is at its proper place, the ZF2:ZF1 and ZF2:ZF2 mutants being unable to perform these reactions [28], [33].

For each zinc finger, one aromatic amino acid, namely F16 in ZF1 and W37 in ZF2, has been shown to be involved in stabilizing contacts with nucleic acids, through stacking interactions with guanines and in some cases thymines [23], [24], [26], [34]–[36]. Moreover, mutational studies of these residues have shown their essential role in nucleic acid binding [12] and chaperone activity [32], [37], [38].

Atomic details of NC binding modes to nucleic acids were determined from the structure of six complexes that were analyzed at high resolution using NMR methods [23], [24], [26], [34]–[36]. Strikingly, in all the complexes one guanine is inserted inside the hydrophobic pocket of ZF2 and stacks extensively with W37. F16 in ZF1 stacks either strongly with guanines (in the case where two guanines are available in the nucleic acid sequence) or stacks partly with a thymine or cytosine residue that remains outside of the hydrophobic pocket of the ZF [25], [26], [35], [36]. Thus, examination of these complexes suggests a strong stabilizing interaction with W37. Moreover, several nucleic acid binding studies with W37A and F16A mutants showed that W37 replacement is much more deleterious than F16 replacement for NC binding [12], [32]. Similarly, deletion of ZF2 is largely more critical than deletion of ZF1 for the binding of the NC domain of Gag to its nucleic acid targets [39]. However, in contrast with these data, the isolated ZF1 was reported to bind DNA and RNA sequences more avidly than the isolated ZF2 [22], [40], [41], while the ZF2:ZF1 and ZF1-ZF1 mutants were found to bind with higher affinity to nucleic acid substrates than the wt and ZF2-ZF2 mutant [42]. This latter result suggests that, either in its isolated form or at the C-terminal position of NC, ZF1 is associated with a higher affinity for nucleic acids than ZF2. It is therefore intriguing that ZF1 appears more important than ZF2 for NC chaperone activity and the viral replication, but not for nucleic acid binding, at least in the context of the wt protein.

This intriguing observation urges us to reconsider the division and coordination of activities between the two ZFs, to get a better understanding of the molecular mechanisms involved in the biological functions of this key HIV-1 protein. Internal dynamics of nucleic acid binding proteins are essential for nucleic acid recognition [43]–[47] and efficient coordination of their various activities. Since these dynamics can be investigated through NMR measurements of ^15^N or ^13^C relaxation times [48]–[53], we reinvestigated the internal dynamics of NC, by ^15^N nuclear relaxation measurements using two fields (950 and 500 MHz). Detailed analysis of the dynamic parameters of the linker residues shows that the two ZFs move relative to each other around a molecular hinge, localized at a crucial position in the linker. In contrast, several other residues in the linker appear quite rigid and in close contact with ZF1. These specific dynamic properties are likely important for controlling the activities of the two ZFs. Interestingly, reexamination of various 3D structures of NC:nucleic acid complexes allowed us to provide a structural basis for the role of ZF1 in the destabilization of secondary structures. Taken together, our data lead to propose a model of coordinated activity between the two ZFs with a critical role for the linker.

## Material and Methods

### Expression and purification of recombinant HIV-1 protein

The recombinant 55-residue NC protein (HIV-1 strain NL4-3) was overexpressed in a (^15^N^13^C)-labeled medium and purified as previously described [19].

### Sample preparation

NMR buffer (25 mM deuterated sodium acetate pH 6.5, 25 mM NaCl, 0.1 mM ZnCl_2_, 0.1 mM 2-mercaptoethanol) was deoxygenated by sparging with argon for 15 minutes. For NMR experiments, 50 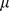
L ^2^H_2_O were added to 0.5 mL of sample into a 5 mm NMR tube. Typical samples contained 1 mM protein.

### NMR data collection and analysis

#### NMR Assignments

The NH and ^15^N assignments were verified at 283 K using HNCA, HN(CO)CA, HNCO, HN(CA)CO, HNCACB, HN(CO)CACB experiments [54], [55]. These experiments were carried out on a Bruker Avance 950 MHz (TGIR-RMN-THC FR3050 CNRS, Gif sur Yvette, France) equipped with cryoprobe triple-resonance with z-axis field gradient.

#### ^15^N NMR relaxation measurements

NMR experiments were carried out on a Bruker Avance 950 MHz and on a Bruker Avance 500 MHz with triple-resonance probe with z-axis field gradient. All measurements were carried out at 283 K. Standard pulse sequences were used for ^15^N relaxation measurements [56]–[58]. In the following, we indicated the parameters used for the experiments recorded at 950 MHz. For experiments at 500 MHz, similar sets of adapted parameters were used. The ^15^N-^1^H correlation experiments were recorded with 1828×256 points and spectral widths of 12 and 34 ppm in ^1^H and ^15^N dimensions, respectively. All the experiments were recorded with a repetition delay of 4 s between successive scans. The T1 data were collected using 20, 50 (repeat), 80, 100, 150, 200, 250, 300, 400 (repeat), 500, 700, 800 and 3 000 ms for the recovery delay. CPMG pulse trains were used with a 0.9 ms delay between successive ^15^N 180° pulses and a ^1^H 180° pulse was applied at the center of the CPMG train to remove cross-correlation between ^15^N CSA and ^1^H-^15^N dipolar interactions [57]. The T2-CPMG data were collected with delays of 16, 32, 64, 80, 128 (repeat), 160, 208 (repeat), 256, 320 and 400 ms. In all experiments, the points corresponding to different relaxation delays were acquired in an interleaved manner.

Exclusively for experiments recorded at 500 MHz the CPMG experiments were repeated with the ^15^N carrier positioned at 103, 116, 120, 129 ppm in order to take into account the errors arising from large resonances [45], [59], [60].

The NMR relaxation data were processed with cosine squared apodization and zero filled once in ^1^H dimension and twice in ^15^N dimension using NMR Pipe. The data were analyzed using SPARKY to determine the relaxation rates and the associated errors.

### Quantitative Analysis of relaxation data

Calculation of the overall rotational diffusion tensor is possible since, in the absence of internal motions, the intrinsic ^15^N relaxation rates depend on the orientations of the N-H bond vectors relative to the axis of the diffusion tensor [52], [61]. The determination requires the T1/T2 ratios, these latter are, to a good approximation independent of internal motions and of the magnitude of the chemical shift anisotropy [61]. However, it is necessary to exclude residues exhibiting motions with timescales greater than several hundred of picoseconds. These latter residues are identified by the fact that they exhibit lower than average ^15^N-{1H} nOes [62]. Similarly, residues involved in conformational exchange in the slow regime could also affect the T1/T2 ratios and have been therefore excluded following guidelines presented in previous works [61]. The programs R2_R1 diffusion and Quadric diffusion (AG Palmer) were used to determine the parameters related to the diffusion tensor, including the correlation time. The improvement associated with the use of a more complex model of diffusion (anisotropic, six parameters) relative to simple models such as isotropic (1 parameter) and axially symmetric (four parameters) models was not statistically significant and data related to this model are therefore not presented.

In a second step, the results were evaluated using the “Model-Free” approach [63] using ModelFree [64]. A brief recall of the formalism of the “Model-Free” approach needs to be presented here. ^15^N T_1_, T_2_ and ^15^N-{^1^H} nOe values are related to the spectral density J(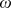) that are determined by the reorientational dynamics of the N-H bond vector [65]. Spectral density terms are the Fourier transforms of the autocorrelation functions of the molecular motions in the time domain.

If the various internal motions are not correlated with the isotropic overall molecular rotation (and this could be questionable as the relative interdomain motion could be, in particular cases, correlated to the overall motion), the total correlation function C(t) can be expressed as
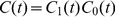
(1)


C_1_(t) is related to the internal motions and C_0_(t) to the overall molecular rotation. In the original Model-Free formalism the internal motions are characterized by the time constant 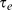 and the generalized order parameter S^2^ and no assumptions are made on the nature of the motions; the autocorrelation function is given by
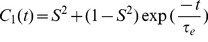
(2)and the corresponding Model-Free spectral density is 
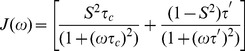
(3)where 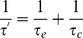
and 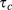 is the overall rotational correlation time and 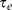 is the correlation time of the fast internal motions. This case is described in the ModelFree approach by two models: (i) the model 1 for which the time scale is extremely fast and tends towards zero (only one parameter S^2^ is determined by the program to describe the internal motion) and (ii) the model 2 in which the time scale of the internal motion is fast but does not tend towards zero (two parameters are used to describe the internal motion 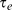 and S^2^).

When internal motions of significant amplitude occur both on fast and slow time scale, a procedure called the “extended Model-Free” approach could be used [66]. In this approach the corresponding autocorrelation function is given by:
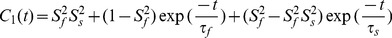
(4)with 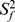
and 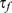 being related to the fast internal motion and 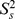
and 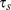 to the slow internal motion. The corresponding spectral density function is described by
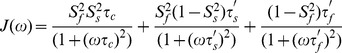
(5)where 
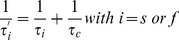


With the ModelFree program [64] in the case where the 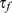 tends towards zero (very fast motion) and therefore the third term in (5) drops, the preceding “extended Model-Free” is described with the model 5 to extract 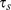, 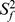 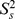.

Although the preceding description is correct for isotropic motion, in the case of more complex motions such as anisotropic motions, more complete information should be found elsewhere [49], [62], [67]

The estimation of the overall motion parameters has been determined for the whole protein using R2_R1 diffusion or ModelFree as described above. We performed calculations using a global correlation time and a diffusion tensor, as it is classically done [64]. The data obtained at 950 MHz and 500 MHz were fitted separately at each field or simultaneously. As previously described, the relaxation rates of each residue were fitted to five models of increasing complexity [45], [64], [68]. The spectral densities J(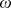) were calculated using as parameters, a uniform value d_CSA_ of −172 ppm for chemical shift anisotropy (CSA) and a value of 1.02 Å for the N-H bond length [69]. In our treatment the standard Model-Free analysis considers only the dipolar ^15^N-^1^H and the ^15^N CSA relaxations pathways. The ^15^N-^13^C' and ^15^N-^13^C 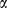 that present measurable contribution are not taken into account because they are predicted to be within the experimental error and anyway less than the influence of all others protons (except the ^1^H_N_ of the same peptide plane) that are themselves usually not taken into account in relaxation studies [70], [71].

## Results

### ^15^N backbone measurements at two fields

T1, T2 ^15^N relaxation times and ^15^N-{^1^H} nOe data obtained at 500 and 950 MHz are presented in Figure S1 in File S1 and in Figure 1, respectively. Experiments at 950 MHz allowed us to obtain a better resolution and to perform more accurate measurements on several residues. Note however that some overlaps remain: namely R52/K34/V13 ^1^H-^15^N cross-peaks cannot be individually resolved. The N- and C-terminal parts are highly flexible as shown by the heteronuclear nOe values less than 0.65 at 950 MHz and less than 0.5 at 500 MHz (Figure 1B-c and Figure S1 in File S1). In comparison, the ZFs appear highly folded with a mean value of 0.66 (500 MHz) and 0.8 (950 MHz) for heteronuclear nOe. These nOe values are in agreement with the T2 values that are weak in ZFs while the terminal parts display high values, indicating highly and poorly folded structures, respectively (Figure 1B-b). Concerning the linker residues (29–35), most of these residues exhibit T1, T2 and nOe values close to those of the two ZFs, however G35 residue differs with significantly lowest nOE value and highest T2 values (relatively to the other residues of zinc finger parts). This residue differs from the other residues of the linker by its nOe and T2 values at the two fields, which are intermediate between those of the ZFs and the terminal flexible parts.

**Figure 2:**
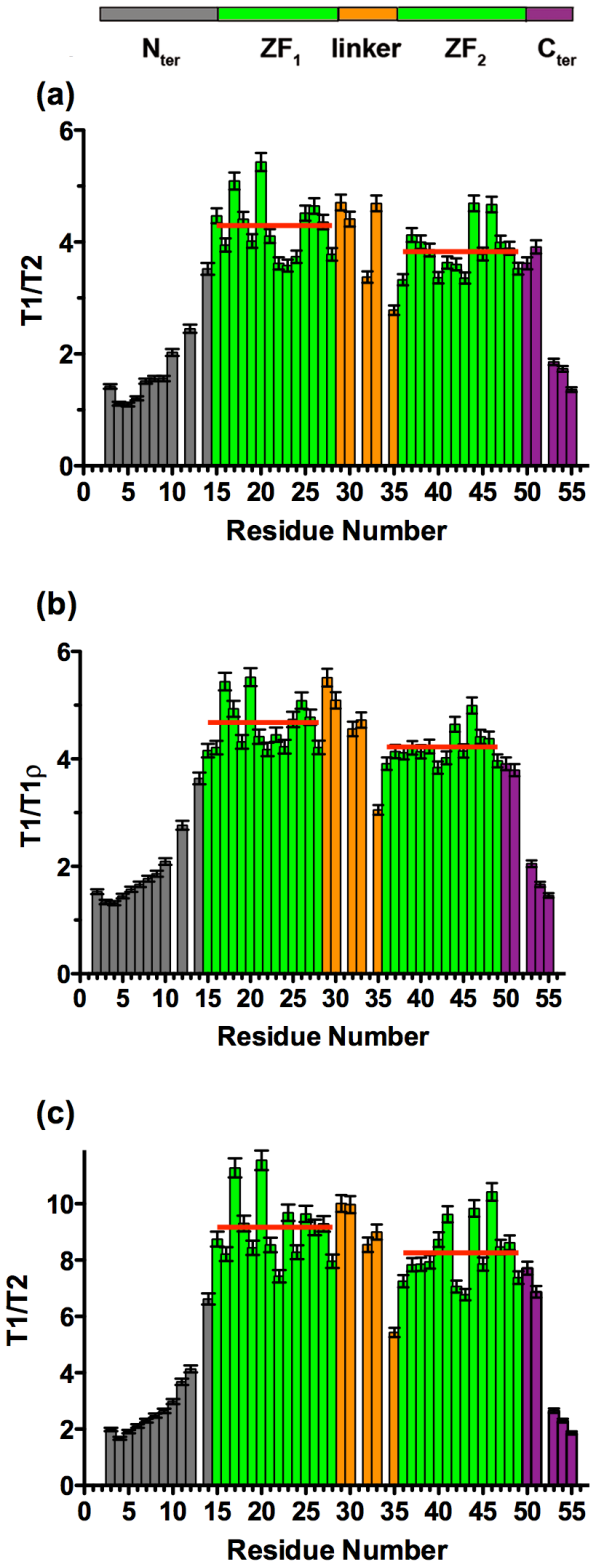
Experimental T1/T2 (or T1/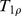) ratios obtained at 500 MHz (this work) **(a)**, 600 MHz [52] **(b)**, 950 MHz (this work) **(c)** for NC at 10°C. At each field, the average value for each zinc finger (ZF1 and ZF2) is indicated as a red bold line. For ZF1, the values for residues 15 to 28 are considered, while for ZF2, residues 36 to 49 are considered.

Analysis of the T1, T2 and nOe values show that while T2 and nOe values are close for the two ZFs (at the two fields), a significant difference is observed at 950 MHz in the T1 values of the two ZFs (average values of 812 ms for ZF1 and 718 ms for ZF2). The difference is 12% at 950 MHz and only 6% at 500 MHz. This is also observed in the T1/T2 ratios (Figure 2) at 500 MHz and 950 MHz (this study) and 600 MHz (in another study [52]). The T1/T2 ratio is particularly interesting because it provides a good estimation of the rate at which each N-H vector reorients with global tumbling [56], [61], [72]. These differences strongly suggest a difference in the rotational correlation times and/or in the diffusion tensors of the two ZFs [73]. The difference between the two ZFs were hardly detectable at 500 MHz but, due to variations in the spectral densities at 950 MHz, the small differences in the two ZF parameters and namely in their correlation times are fully observable at this higher field.

To further explore the subtle differences between the values for the ZFs and the different residues of the linker, a more quantitative analysis of the data is needed. We performed several analyses, namely an analysis of the diffusion tensors and of rotational correlation times [52] and a model free analysis to determine the order parameters [64].

### Quantitative analysis of the data: determination of the rotational diffusion tensors

First, we used the R2R1 diffusion program to calculate the diffusion tensor from the ^15^N R2/R1 experimental data at the two fields. The data were analyzed by considering the two ZFs, either separately (ZF1, ZF2) or together (ZF1+ZF2). As already described, the T1/T2 ratio provides a measure to determine the rate at which the NH bond reorients as a result of global tumbling [61]. The procedure needs however to exclude the residues with lower heteronuclear nOes and those that could be affected by chemical exchange [61], [68]. Additionally, we exclude the linker (29–35) residues in order to consider only data from ZFs. The results from fitting procedures are presented in Table 1. The data obtained at two different fields converge to show that: (i) a better fit to experimental data is obtained when data from the two ZFs are considered separately; (ii) the ZF1 correlation time is significantly larger than the ZF2 one; (iii) the data from ZF1 fit better with a axially symmetric anisotropic diffusion tensor while the ZF2 one appears isotropic (the 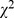 appears lower with the axially symmetric model, the F statistic associated to the use of a more complex fully anisotropic model is not significant).

**Table 1:** Diffusion parameters calculated for the ZF1 and ZF2 of NC calculated with relaxation data obtained at 500 and 950

Data	Model	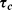 (ns)	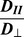	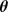 (deg)	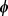 (deg)	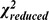	F
**950 MHz**							
ZF1+ZF2	Isotropic	5.44	1.00			9.50	-
	Axially symmetric	5.44	1.19	44	20	9.00	1.37
ZF1	Isotropic	5.66	1.00			5.93	-
	Axially symmetric	5.70	1.27	41	21	3.19	3.80
ZF2	Isotropic	5.25	1.00			4.18	-
	Axially symmetric	5.25	1.18	26	23	2.66	1.69
**500 MHz**							
ZF1+ZF2	Isotropic	6.36	1.00			10.42	-
	Axially symmetric	6.36	1.22	33	19	10.42	1.05
ZF1	Isotropic	6.58	1.00			11.52	-
	Axially symmetric	6.66	1.54	40	21	4.71	6.70
ZF2	Isotropic	6.17	1.00			5.33	-
	Axially symmetric	6.15	1.18	39	21	5.02	1.23

The parameters have been calculated with the two ZFs taken together (ZF1+ZF2) or separately. D_II_ and D⊥ are the parallel and perpendicular components, respectively, of the diffusion tensor in the case of an axially symmetric model. 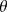 and 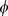 angles define the orientation of the main axis of diffusion tensor in spherical coordinates. F is the F-statistic associated to the 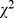 test and evaluates if the gain associated with the use of a model with more variables (axially symmetric) relative to the simple (isotropic) model is significant.

In a second step, we included the data of the linker residues to the set of data of the two ZFs measured at the two fields. We first added the data concerning the 30–33 residues (A30, R32, K33) either to the data of ZF1 or to those of ZF2 (Table 2). In a third step, we further added the data concerning the G35 residue. This two-step strategy was motivated by the very peculiar behavior of the G35 residue, which exhibited low heteronuclear nOe and high T2 values at the two fields. The data in Table 2 show again a good agreement between the values obtained at the two fields. Moreover, we observe that: (i) addition of the 30–34 residues to ZF1 slightly worsened the fit at 500 MHz (comparing the 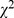 values in second and fourth lines of the first part (500 MHz) of Table 2) but improved the fit at 950 MHz (comparing 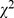 values in the second and fourth lines of the second part (950 MHz) of Table 2); (ii) addition of the same residues to ZF2 entailed poor fitting at the two fields and (iii) further addition of G35 considerably deteriorated the fit of both ZFs. This suggests that the dynamics of the 30–34 residues are similar to the dynamics of ZF1, while a clear discontinuity exists with the G35 residue that exhibits very peculiar dynamic properties relative to the other linker residues.

**Table 2:** Diffusion parameters calculated for the two zinc fingers of NC at 500 and 950

Data	Model	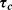 (ns)	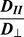	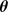 (deg)	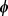 (deg)	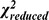
**500 MHz**						
ZF1	Isotropic	6.58	1.00			11.52
	Axially symmetric	6.66	1.54	40	21	4.71
ZF1+link 30–34	Isotropic	6.58	1.00			14.00
	Axially symmetric	6.66	1.53	16	19	7.35
ZF1+link 30–35	Isotropic	6.51	1.00			24.84
	Axially symmetric	6.51	1.70	22	36	16.60
ZF2	Isotropic	6.17	1.00			5.33
	Axially symmetric	6.15	1.18	39	21	5.02
ZF2+link 30–34	Isotropic	6.24	1.00			10.65
	Axially symmetric	6.26	0.88	6.	7	12.62
ZF2+link 30–35	Isotropic	6.12	1.00			17.37
	Axially symmetric	6.17	1.17	24	25	20.46
**950 MHz**						
ZF1	Isotropic	5.66	1.00			5.93
	Axially symmetric	5.70	1.27	41	21	3.19
ZF1+link 30–34	Isotropic	5.68	1.00			5.74
	Axially symmetric	5.72	1.26	17	38	2.56
ZF1+link 30–35	Isotropic	5.48	1.00			31.79
	Axially symmetric	5.59	1.48	7	34	29.03
ZF2	Isotropic	5.25	1.00			4.18
	Axially symmetric	5.25	1.18	26	23	2.66
ZF2+link 30–34	Isotropic	5.35	1.00			9.14
	Axially symmetric	5.35	1.15	37	28	9.63
ZF2+link 30–35	Isotropic	5.20	1.00			24.63
	Axially symmetric	5.32	1.22	23	16	35.29

See Table 1 for informations on the presented parameters.

### Fitting of the ^15^N relaxation data with Model-Free

From the above data a very important point emerges. Indeed, while the respective behaviors of the two N- and C-terminal domains are similar at the two fields, their correlation time 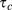 strikingly differ at 500 and 950 MHz. Moreover, if we also examine the data obtained in the same conditions but at 600 MHz in a preceding work [52] the values are intermediate. Therefore the overall rotational correlation time appears to be the clearly field-dependent showing decreasing values as the field increases. This behavior is typical of proteins exhibiting slow internal motion [62], [74]. Therefore in the following we use the ModelFree program to analyze the effect of the inclusion of a slow motion on the apparent correlation time and on the data fitting.

A brief recall of the formalism used in the “model-free” approach [64], [75] has been presented in the Materials and Methods section. The inclusion of a slow motion with the Model-Free approach uses the “extended model free approach” that is made possible through the model 5 described in Materials and methods [64]. In this model the generalized order parameter of the slow and fast motion (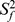 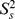) and the correlation time of the slow motion are determined in addition to the overall correlation time 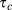. The calculations have been made for several models (models 1, 2 and 5) in order to evaluate the effect of the inclusion of a slow motion in model 5. The results are presented in Table 3. The calculations have been made for the residues that are clearly exempt of chemical exchange and of large-amplitude motions on a time scale longer than a few hundred picoseconds (which can be identified on the basis of a lower nOe value) using the criteria described previously [61]. Note that selected residues are not exactly the same as those used in Table 1 because here the selection criteria for the different residues must be matched for the data at the two fields. Using this procedure 18 residues have been selected.

**Table 3:** Results from analysis of NMR relaxation data obtained at 950, 500 and 600([52]) using various models of motions with the Model-Free analysis.

Domain	Data (MHz)	Model	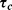 (ns)	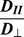	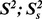 _ (a)_	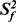	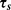 (ps)	E/N
ZF1	950	1	5.7	1.3	0.91			1.6
ZF2	950	1	5.3	1.17	0.94			1.0
ZF1	500	1	6.6	1.6	0.78			0.9
ZF2	500	1	6.2	1.25	0.79			1.2
ZF1	500, 600, 950	1	5.9	1.7	0.83			7.2
ZF2	500, 600, 950	1	5.5	1.23	0.85			6.2
ZF1+ZF2	500, 600, 950	1	5.7	1.3	0.84			9.8
ZF1+ZF2	500, 600, 950	2	6.1	1.6	0.81		238	5.9
ZF1+ZF2	500, 600, 950	5	8.3	1.4	0.64	0.84	2230	1.2

The different parameter and model of motions are described in the text. The results have been obtained using Powell optimizations in Model-Free, the S^2^ and 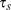 values are the average values of the different residues contained in N (ZF1) and C (ZF2) domains. The selected residues are those that contain at both fields neither signs of conformational exchange neither large amplitude, long time scale internal motions (characterized by low heteronuclear nOe). In (a) the relevant parameter for model 1 is S^2^ and those relevant for model 5 (that contained both a fast and slow component) is 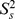. The 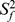 value is related to the fast motion described by the model 5, recall that with ModelFree, we cannot extract the value of the correlation time of the fast motion as it is supposed to be extremely fast. See Table 1 for information on the presented parameters. E/N is the residual 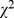 error per residue.

We used the first four lines of Table 3 to check that the correlations times and the 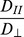 parameter found with ModelFree are the same than with the preceding approach (R2_R1 diffusion program and data presented in Table 1). We clearly see that in these four cases, the residual error per residue (E/N) is quite low indicating a good fitting of the data at each field. However, when the data at the three fields (including the data of Lee et al. (1998)[52]) are fitted simultaneously with model 1 the resulting error is very high (see Table 3 the E/N (residual error per residue) values: 7.2 for ZF1 and 6.2 for ZF2). Including the “extended Model-Free” approach with the model 5, the residual error per residue (E/N) drops dramatically (E/N  = 1.2, last line of Table 3) underlining the validity of the hypothesis of a slow motion occurring in the nanosecond time scale. The calculations with model 5 (in which the internal motions are described by both slow and fast motion) have also been made in different conditions, namely by changing the maximum value that the correlation time of the slow internal motion can take (Table S1 in File S1). We show that the maximum value is close to the range of 2–3 ns. We also observe the change of the apparent overall correlation time 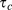 with the variation of the correlation time of the slow motion (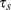) as previously described [62], [74]. Interestingly the 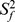
values are close to 0.85, a reasonable and typical value for fast librational motion in proteins. The 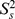
values are close to 0.64 indicating the strong involvement of the slow motion in the global motion features of the protein. When these values are considered for each domain separately, a striking difference is found: 0.66 for ZF1 and 0.60 for ZF2 indicating that the C-domain could be more flexible that the N-domain. All the values related to the slow motion are close to those obtained in a precedent work on a similar motion occurring in calmodulin [62].

The existence of this slow motion complicates seriously the determination of the internal motional parameters for each residue as the Model 5 is the most complex model that can be extracted with the ModelFree program and thus cannot take into account additional exchange processes or motions occurring at intermediate time scale (several hundreds of picosecond) [74]. These two last categories of motions are very common in internal dynamics of proteins. A complete extraction of all the parameters describing the various motions in our system is thus clearly too complex and beyond the scope of the present study. Interestingly, a partial description of the protein dynamics can be provided by performing a classical Model-Free approach on the data obtained at each field (950 and 500 MHz). This approach is qualitative since it does not take not into account the slow internal motion identified above. However, it can reasonably describe the exchange and intermediate scale motions of the individual residues. Note that if data at only one field (950 or 500 MHz) was obtained, this approach would have appeared perfectly sufficient.

**Figure 3:**
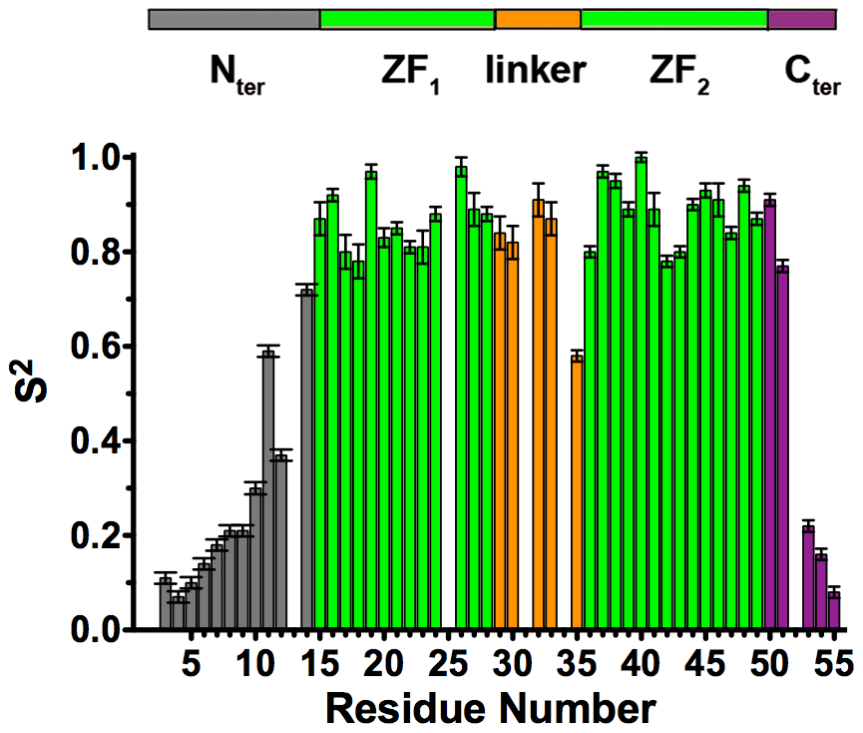
S^2^ order parameters determined from Model-Free analysis of ^15^N relaxation data obtained at 950°C.

Thus, the T1, T2 ^15^N relaxation times and the ^1^H-^15^N heteronuclear nOe at only one field (either 500 or 950 MHz) were used to extract the internal motion parameters for each residue using the Model-Free approach. In this approach, the relaxation data for each residue were fitted with five internal motions of increasing complexity as described previously [45], [64] and the model giving the best fit was assigned to each residue. Most of the residues could be fitted with Model 2, 4, 5 while only a few residues could be fitted with Model 1 and 3 (Materials and Methods). The residues of the N- and C-terminal flexible regions could be only fitted with Model 5 while most of the ZF residues were fitted with Model 2 or 4. The order parameters extracted from the relaxation data at the two fields are presented in Figure 3 (950 MHz) and in Figure S2 in File S1 (500 MHz). The results for the two fields are qualitatively close to each other but fewer points are obtained at 500 MHz due to overlap or impossibility to fit the data with the simple models available in ModelFree. The existence of regions of different flexibility emerges well from the data and is globally in agreement with precedent works [52], [53], where the order parameters were not explicitly extracted for all residues. The residues of the N- and C-terminal domains exhibit low values for the order parameter from 0.1 to 0.5, while the two ZFs appear highly structured with values from 0.67 to 0.81 at 500 MHz and 0.77 to 1.0 at 950 MHz. The two ZFs appear equally structured, with order parameter values that are not significantly different. For the linker residues, the order parameter values for A30, R32 and K33 are similar to those of the ZF residues while a significantly smaller value is obtained for G35 (0.44 at 500 MHZ, 0.58 at 950 MHz). This last value is intermediate between the values observed for the ZF residues and those of the flexible C- and N-terminal domains, revealing a significant flexibility of this residue. It is important to note the discontinuity between the G35 value and the values of the other residues in the linker. This clearly suggests that G35 is a hinge allowing the two ZFs to move in respect to each other. Note that we have described thoroughly this internal motion in the preceding paragraphs and that we have found it to occur in the nanosecond timescale (2–3 ns).

### Additional Characterization of the protein conformation

We analyzed in detail the 2D NOESY spectra recorded at 950 MHz focusing our investigations on the nOes interactions between residues belonging to the two ZFs fingers and the linker domain. Since interknuckle nOes have been reported in several studies [26], [52], [76], we checked for their existence in our spectra. We observed most of the nOes already described (A25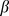-W37
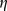
2; A25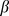-W37
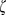
2, A25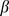-W37
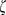
3; W37
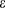
3-F16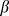; W37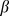-F16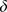; etc…), but could not find nOes between the aromatic protons of F16 and the aromatic protons of W37. Concerning the linker residues, a very large number of nOes (more than 25) were observed between several protons of N17 (and mainly the two amino protons resonating at 6.81 and 8.52 ppm in our conditions) and the linker residues. Some of these nOes are rather strong and involve interactions with C28, R29, A30, P31, R32, K33 residues, revealing therefore an extended network of contacts. While some of these nOes have been observed previously [76], such a large network of contacts involving linker residues has never been underlined. However, it was not necessary to build new structures taking into account these nOes as the existing structures (1mfs and 1esk) are in agreement with most of these nOes. The others nOes contacts involving the W37
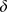
1, 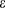
1 and 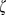
2 protons and the R32, K33 residues have been already described.

Globally, our data suggest the insertion of the N17 residue inside the turn of the linker induced by the P31 residue (Figure S3 in File S1). Note also that this insertion appears to induce a very large chemical shift difference between the two N17 amide protons (1.71 ppm). This difference is much larger than that usually observed for Asn amide protons and for others Asn residues of NC. It indicates that the N17 amide protons experiment shielding or local field effects. Hydrogen bonding could explain theses effects. The nOe contacts between the F16 and W37 residues of the two different zinc fingers indicate that the amplitude of the motion between the two domains is limited in our conditions (10°C); in line with the limited size of the hinge (G35 residue) that constraints the amplitude motion.

### Examination of the three-dimensional structures of NC-nucleic acids complexes

**Figure 4:**
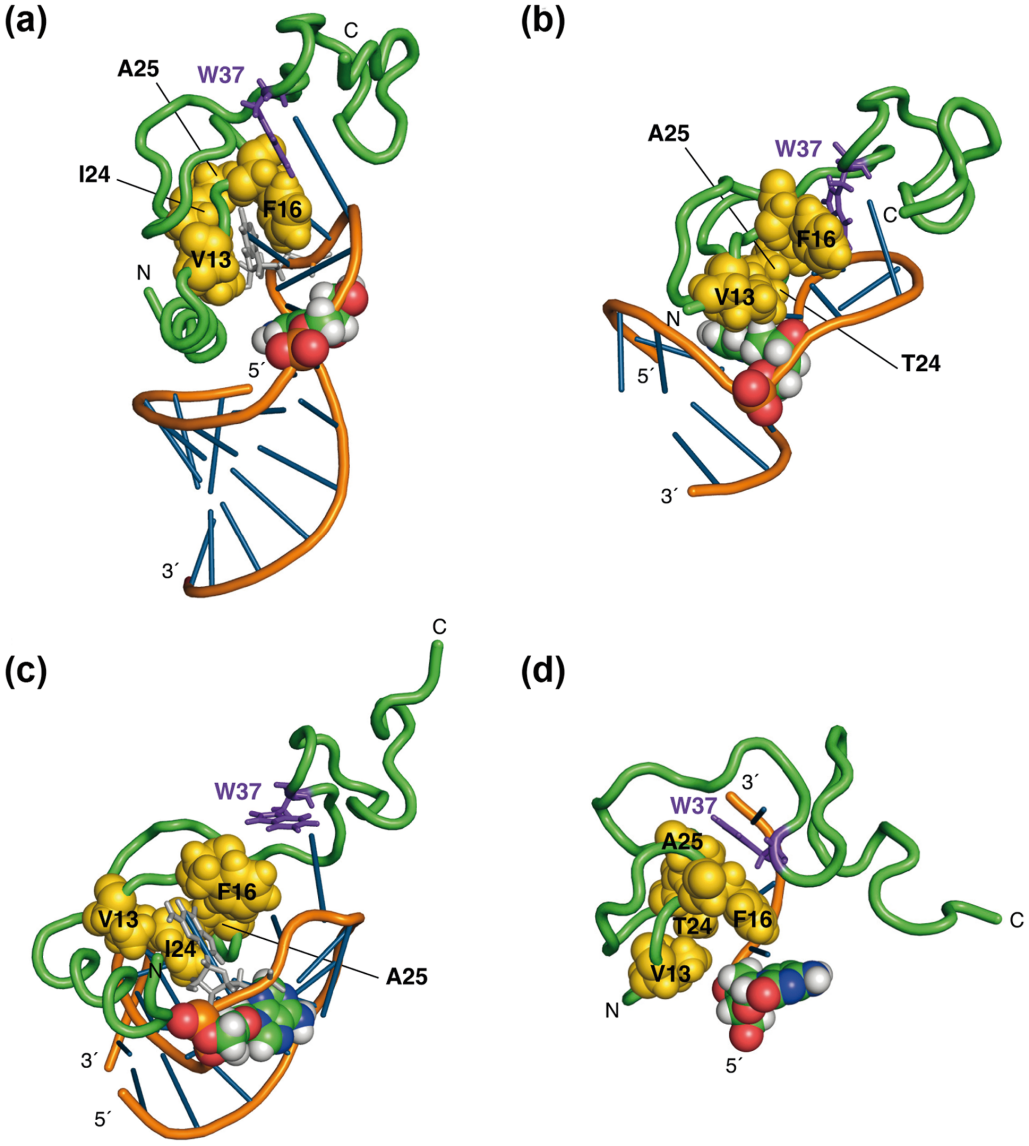
Views of four NC-oligonucleotide complexes. **(a)** SL3-NC (pdb 1a1t); **(b)** PBS-NC (2exf); **(c)** SL2-NC (1f6u); **(d)** mini-cTAR-NC (2l4l). For each complex, the oligonucleotide and protein backbones are underlined in orange and green, respectively. The protein side chains are not shown except for W37 (stick in violet) in ZF2, and V13, F16, A25, I/T 24 in ZF1 (spheres in yellow). Additionally, the stem residue closest to the loop is shown as a sphere (color mode by atom type). In **(a)** and **(c)**, the guanine bound to ZF1 is indicated in stick and light grey

We investigated carefully the four three-dimensional structures of NC bound to stem-loops that are available in the PDB (Figure 4). In all of these complexes, NC is bound to the unpaired region. We examined more particularly the position of the two ZFs relative to the stem base pairs adjacent to the loop. In all the complexes, ZF2 (with W37 in violet and stick mode) is positioned farther from the stem than ZF1. Furthermore, one guanine of the loop is inserted in ZF2 in all the complexes, while another guanine is inserted in ZF1 only when two unpaired guanines are present in the loop. This strongly suggests that ZF2 possesses a stronger affinity for guanines than ZF1 as already mentioned in the introduction section.

**Figure 5:**
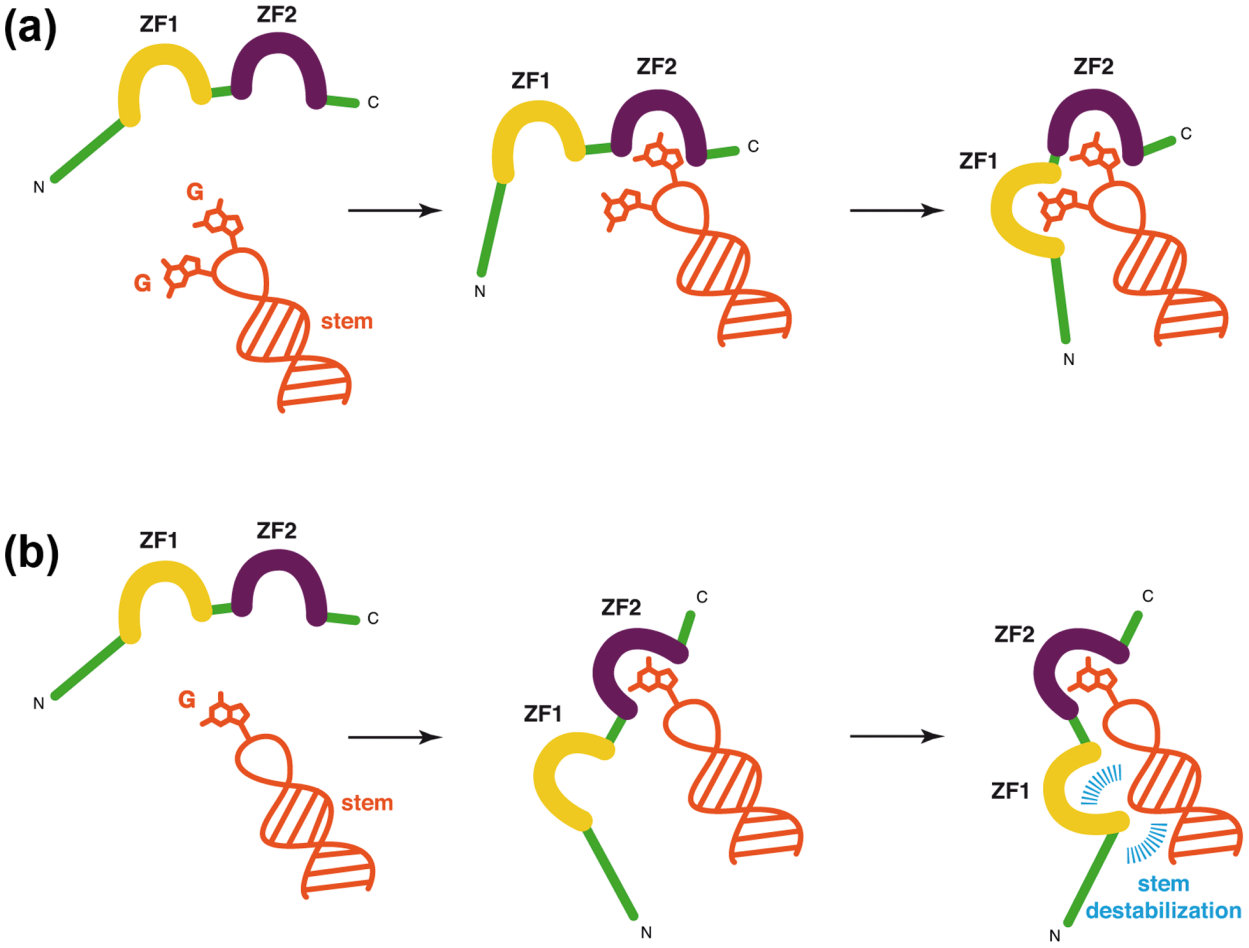
Schematic representation of the task specialization of each ZF upon binding to nucleic acids. The nucleic acid fragment is in orange, NC zinc-fingers are in yellow (ZF1) and purple (ZF2), and the rest of the protein is in green. (**a**) Situation #1 with two accessible guanines. ZF2 binds first to an accessible guanine, and ZF1 binds the remaining guanine. The stem is not destabilized. (**b**) Situation #2 with only one accessible guanine. ZF2 binds first to this accessible guanine, and ZF1 remains free to contact the stem via its large hydrophobic platform. The stem is destabilized (in blue).

ZF1 exhibits an extended hydrophobic platform formed by the residues V13, F16, I/T24, A25 (shown as yellow spheres in Figure 4). In Figure 4, we highlighted the positioning of this hydrophobic platform relative to the closest residues of the stem. In SL2 and SL3, one guanine (located in the apical loop and indicated by light grey in Figure 4a and c) is inserted in the ZF1 hydrophobic platform that is localized far from the stem. In contrast, in the complexes with (-) PBS and mini-cTAR (Figure 4b and d) in which ZF1 does not interact with a guanine, ZF1 contacts one base and one sugar of the stem. These observations suggest that the guanine binding to ZF1 mobilizes the residues of the hydrophobic platform and therefore hampers its binding to the stem and thus, prevents the stem destabilization. In contrast, in the complexes in which ZF1 does not interact with guanine, the hydrophobic platform could contact the stem and destabilize it (Figure 5).

## Discussion

### Dynamic properties of the linker residues

In this study, our data underline the difference of dynamic behavior between the different domains of NC and notably between the two ZFs, as shown by the quantitative analysis of relaxation data (Table 1) as well as by the raw data and particularly the T1 and T1/T2 values at 950 MHz (Figures 1B and 2). Our data suggest the existence of a relative motion of the two domains (N- and C-terminal) around the G35 and perhaps K34 residues of the linker, acting as a hinge. The observed changes in the apparent overall correlation time with the magnetic field are typical of such a slow motion around a defined hinge [62], [74] and can be well modeled using the “extended Model-Free” approach.

After the identification of this slow motion, we use the classical Model-Free approach at one field (either 950 or 500 MHz but not simultaneously) to describe the dynamic status of each residue in the protein. From the order parameter obtained for the linker residues, we could conclude that residues A30, R32, K33 are much less flexible than G35 and share common properties with residues of ZF1, suggesting the existence of a persistence length extending from the structured ZF1 to most of the linker residues [73], [77], [78]. Both this persistence length and the constraining nature of L-proline at position 31 confer rigidity to the linker [76]. In contrast, the seventh residue of the linker, G35 exhibits a significantly lower order parameter indicating that it is highly flexible. Consistent with this notion is the fact that the two H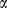 protons of G35 present identical chemical shifts, as it could be expected for mobile residues and as it is the case for residue G4 located in the unstructured part of NC. In contrast residues G19, G22, G40 and G43 located in the structured ZFs display all non-equivalent chemical shifts for their two H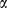 protons. Therefore, our data suggest that the two ZFs move relatively to each other about this pivotal position. Nevertheless, we cannot completely exclude that the K34 neighbor residue, for which the relaxation data could not be measured due to resonance overlapping is also dynamic (see also [53]). However, due to the very small size of the hinge region (one or two residues), the magnitude of the relative motions of the ZFs is probably rather limited. This could explain why a spatial proximity between the two ZFs has been repeatedly found, notably under the form of nOes observed between residues belonging to the two ZFs [52], [76] and FRET between the aromatic residues of the two ZFs [79]. It has been also suggested that the spatial proximity between the aromatic residues could be highly dynamic [52] and that increasing the temperature increases the flexibility of the linker, probably by the fact that K34, K33 and R32 residues became also flexible and therefore increasing the amplitude of the conformational changes and resulting in an “opening” of the protein [53]. Note that slight changes in the linker composition such as the replacement of L-Pro by D-Pro are sufficient to prevent the spatial proximity of the ZFs [76].

Besides these dynamic aspects, we consider with attention the extended network of nOes involving particularly the side chain N17 of ZF1 and several protons of the C28, R29, A30, P31, R32 and K33 residues. These nOes suggest an insertion of a hairpin loop of ZF1 (with N17 located at its extremity) inside the turn of the linker (See Figure S3 in File S1). This folding is found in the two deposited structures (1mfs and 1esk) and is expected to modulate the properties of ZF1 relative to ZF2 explaining the non-symmetrical role of the linker in respect to the two ZFs as well as the differences in the motions of the two ZFs. Our results support the critical role of the linker residues on the NC architecture and activities [76], [80].

### Implication for the nucleic acid binding properties and roles of the two zinc fingers

The analysis of the dynamical behavior performed above revealed the existence of a relative motion of the two zinc fingers around a hinge constituted by the G35 residue at 10°C, and the non-symmetrical contacts of the linker residues with the two ZFs (See Figure S3 in File S1). We suggest that these contacts affect the nucleic acid binding ability of ZF1 relative to ZF2. Indeed in the last years a sufficient number of NC-nucleic acids structures became available, so that it is now possible to draw some general rules. A clear indication from these binding studies is that ZF2 exhibits a higher avidity than ZF1 for unpaired guanines, since in all studied complexes in which only one guanine is present in the nucleic acid binding site, this guanine is inserted in ZF2 [26], [35], [36]. Moreover, the higher affinity of ZF2 for guanine as compared to ZF1 was also confirmed in biophysical studies [12], [32]. Following the “initial” binding of a first guanine residue by ZF2 [3], a reorganization of the linker residues involved in nucleic acid binding (R32, K33, K34) may occur, leading to the insertion of a second guanine inside the highly hydrophobic pocket of ZF1 (in the case where two guanines are present in the bound sequence). However, the sequential nature of such events remains to be demonstrated. This conclusion is further substantiated by the significantly smaller order parameter observed for the C-terminal as compared to the N-terminal ZF (0.60 vs. 0.66) suggesting that the former is more flexible and prone to adapt to unpaired guanines.

Examination of the three-dimensional structure of the published complexes of NC with various DNA and RNA stem-loops provides interesting information on their topologies that complement the dynamic data. In the complexes of NC with SL2 and SL3, in which one guanine is inserted in each ZF, no contact occurs between the hydrophobic residues of ZF1 (V13, F16, A25, T/I24) and the stem (Figure 4a and c). In contrast, with the PBS complex, and to a lesser degree with the mini-cTAR complex, a guanine is inserted only in ZF2 and the hydrophobic platform of ZF1 contacts the stem (especially V13 and T24), suggesting that it can destabilize it. Thus, the binding of a guanine base into ZF1 in the complexes with SL2 and SL3 likely mobilizes the hydrophobic platform of ZF1, so that it is no more available for destabilizing the stem. This observation is in agreement with previous data showing that the SL2 and SL3 sequences, involved in the selective packaging of HIV-1, genome are not significantly destabilized by NC [24], [81]. In contrast, it is necessary that NC destabilize the PBS and cTAR hairpins to favor the PBS(−)-PBS(+) and TAR-cTAR annealing processes that are required for the strand transfer events during reverse transcription [35], [82]–[84] (Figure 5).

In this model, each ZF is specialized in one function. While ZF2 is required for the binding of accessible and flexible guanines, ZF1 is needed either for the binding of a second guanine (if a GXG sequence is present) or alternatively for the destabilization of base pairs through its large hydrophobic platform (when only one guanine residue is present in the bound sequence by example TG in PBS). Due to this specialization, ZF swapping or duplication mutations such as the ZF2-ZF1 or ZF2-ZF2 mutants (associated with poor chaperone activity) could not achieve similar activity as ZF1-ZF2 or ZF1-ZF1 constructs (associated with strong chaperone activity) [28], [32]. Indeed, while in the two last mutants, ZF1 necessary for destabilization is at the right place, this is not the case for the two others mutants (ZF2-ZF1 and ZF2-ZF2), where the ZF at the N-terminal position is ZF2. It is likely that the hydrophobic platform of ZF2 is too small to destabilize base pairs in double stranded nucleic acids.

## Conclusions

In conclusion, ^15^N relaxation studies of free NC show that the two zinc fingers move relatively to each other around a hinge of small size (G35 and perhaps also K34). The dynamical data further indicate that the motion is in the nanosecond range. Except the G35 flexible residue, the linker residues appear rather rigid, and some of them interact with the ZF1 residues. These new results can be used to explain the dissymmetric binding properties of the two zinc fingers. We suggest that the linker-ZF1 contacts modulate the nucleic acid binding properties of the ZF1 relative to the more mobile ZF2. Therefore, ZF2 could be involved in the “initial” binding to exposed guanines, while ZF1 could either bind another available guanine or be involved in the destabilization of the double-stranded part of the nucleic acid substrate depending on the number of guanines in the target sequence.

## Supporting Information

File S1 **This file contains Figures S1 - S3 and Table S1.** Figure S1. Experimental 15N NMR relaxation data (Longitudinal (T1), transverse (T2) relaxation times and heteronuclear nOe) for backbone atoms obtained at 500 MHz for NC at 10°C.
Figure S2. Order parameters determined from Model-Free analysis of 15N relaxation data obtained at 500 MHz for NC at 10°C.
Figure S3. Structure of NC (pdb 1esk) showing the positioning of the N17 side chain (in red) relatively to the linker, the different residues R29 to K33 are shown with their side chain in blue, K34 in green, G35 in yellow. The side chains of the others residues are not shown, the zinc atoms are shown as spheres and the cysteines and histidines coordinating zinc atoms are shown in orange.
Table S1. Results for the fits of the ^15^N NMR relaxation data obtained at 950, 600 ([52]) and 500 MHz using various models of motions with the model-free formalism described in Materials and Methods.
(DOC)
