# Supplementary material for: Dynamics of Linker Residues Modulate the Nucleic Acid Binding Properties of the HIV-1 Nucleocapsid Protein Zinc Fingers
Source: PLoS One. 2014 Jul 16;9(7):e102150. doi: 10.1371/journal.pone.0102150 (PMC4100767; doi:10.1371/journal.pone.0102150)
Supplement: File S1 — This file contains Figures S1 - S3 and Table S1. Figure S1. Experimental 15N NMR relaxation data (Longitudinal (T1), transverse (T2) relaxation times and heteronuclear nOe) for backbone atoms obtained at 500 MHz for NC at 10°C. Figure S2. Order parameters determined from Model-Free analysis of 15N relaxation data obtained at 500 MHz for NC at 10°C. Figure S3. Structure of NC (pdb 1esk) showing the positioning of the N17 side chain (in red) relatively to the linker, the different residues R29 to K33 are shown with their side chain in blue, K34 in green, G35 in yellow. The side chains of the others residues are not shown, the zinc atoms are shown as spheres and the cysteines and histidines coordinating zinc atoms are shown in orange. Table S1. Results for the fits of the 15N NMR relaxation data obtained at 950, 600 ([52]) and 500 MHz using various models of motions with the model-free formalism described in Materials and Methods. (DOC) [file pone.0102150.s001.doc]

**File S1 SUPPORTING INFORMATION**

**Dynamics of linker residues modulate the nucleic acid binding properties of the HIV-1 nucleocapsid protein zinc fingers**

Loussiné Zargarian^1^, Carine Tisné^2^, Pierre Barraud^2^, Xiaoqian Xu^1, 3^, Nelly Morellet^4^, Brigitte René^1^, Yves Mély^5^, Philippe Fossé^1^ and Olivier Mauffret^1*^

^1^ Laboratoire de Biologie et Pharmacologie Appliquée, Ecole Normale Supérieure de Cachan, Centre National de la Recherche Scientifique, Unité Mixte de Recherche 8113, Cachan, France.

^2^ Laboratoire de Cristallographie et RMN Biologiques, Université Paris Descartes, Centre National de la Recherche Scientifique, Unité Mixte de Recherche 8015, Paris, France.

^3^ Department of Life Sciences, East China Normal University, Shanghai, People’s Republic of China.

^4^ Centre de Recherches de Gif, Institut de Chimie des Substances Naturelles, Centre National de la Recherche Scientifique, Gif sur Yvette, France.

^5^ Laboratoire de Biophotonique et Pharmacologie, Centre National de la Recherche Scientifique, Unité Mixte de Recherche 7213, Faculté de Pharmacie, Université de Strasbourg, Illkirch, France.

* To whom correspondence should be addressed. Tel: 33 1 77 40 77 33; Fax: 33 1 77 40 76 71; Email: [olivier.mauffret@lbpa.ens-cachan.fr](mailto:olivier.mauffret@lbpa.ens-cachan.fr); Address: LBPA, ENS de Cachan, CNRS, 61 avenue du Président Wilson, F-94235 Cachan, France.

**
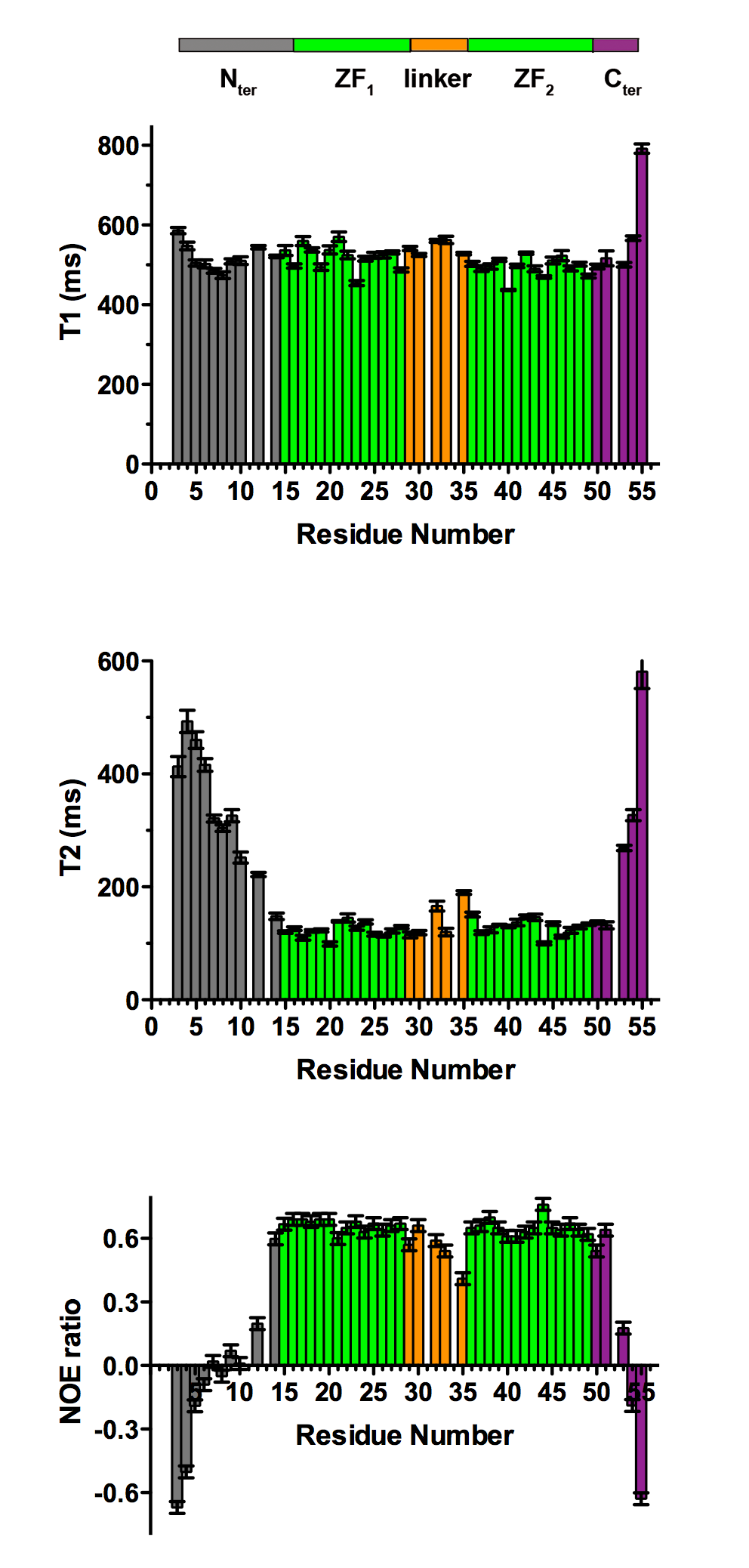
**

**Figure S1. Experimental ^15^N NMR relaxation** (Longitudinal (T1), transverse (T2) relaxation times and heteronuclear nOe) data for backbone atoms obtained at 500 MHz for NC at 10°C.

**
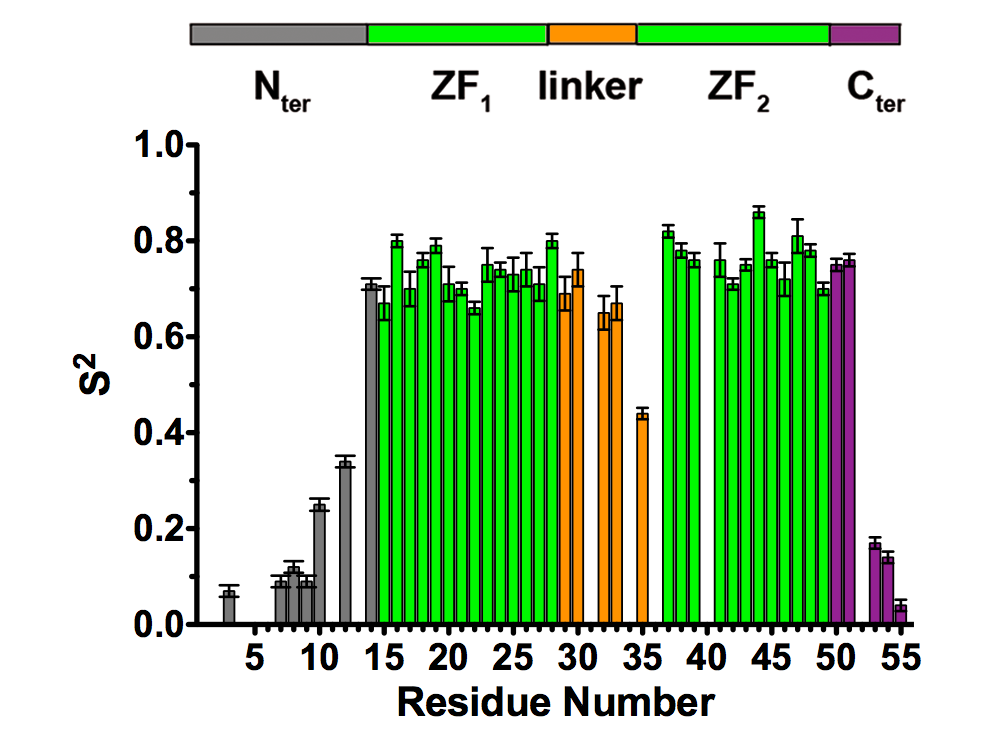
**

**Figure S2**. **Order parameters determined from model-free analysis of ^15^N relaxation** data obtained at 500 MHz for NC at 10°C

**
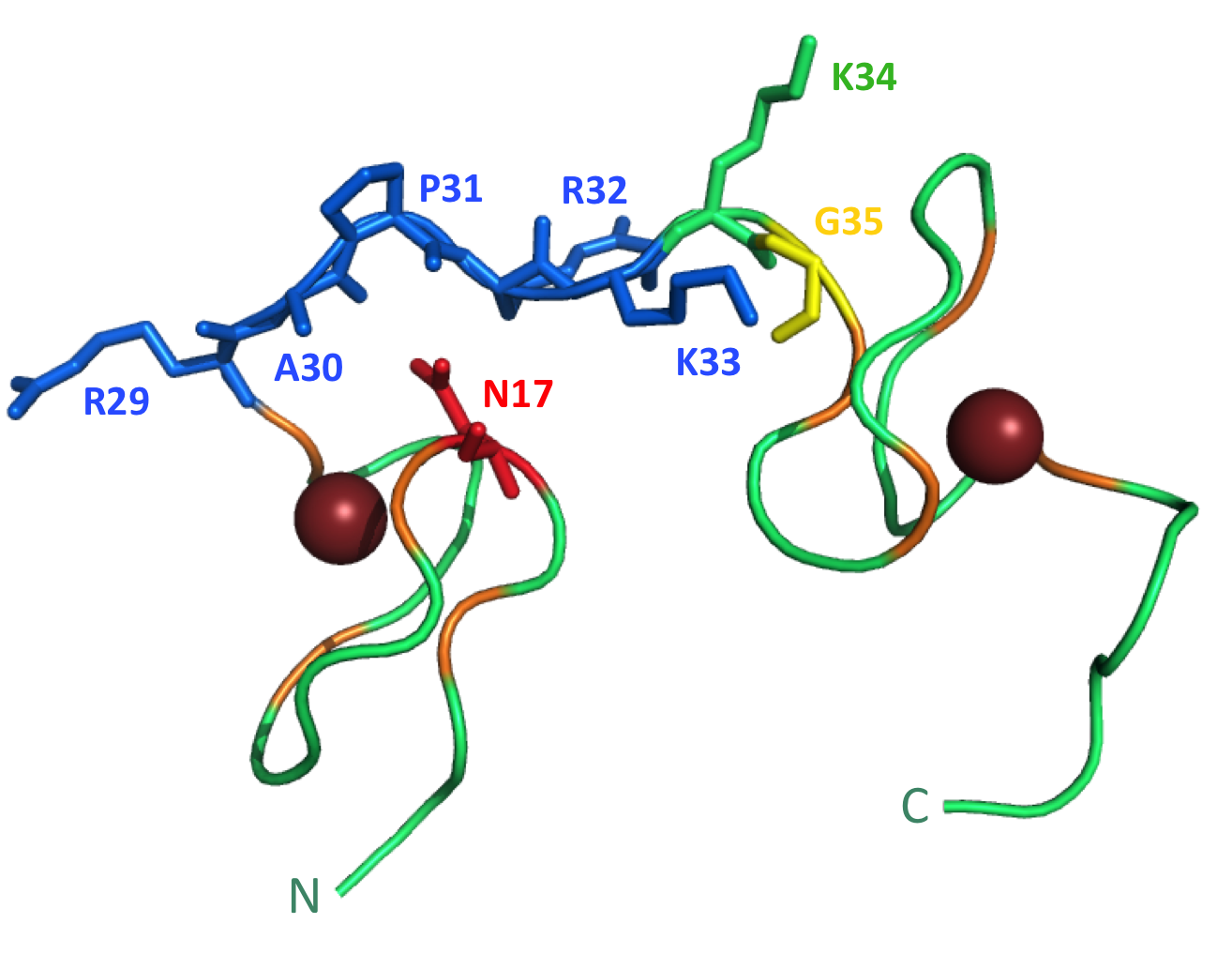
**

**Figure S3. Structure of NC** (pdb 1esk) showing the positioning of the N17 side chain (in red) relatively to the linker, the different residues R29 to K33 are shown with their side chain in blue, K34 in green, G35 in yellow. The side chains of the others residues are not shown, the zinc atoms are shown as spheres and the cysteines and histidines coordinating zinc atoms are shown in orange.

Supplementary Table S1

Results for the fits of the ^15^N NMR relaxation data obtained at 950, 600 (Lee et al., 1998) and 500 MHz using various models of motions with the model-free formalism described in Materials and Methods.

| **Domain N+C** | **Data (MHz)** | **τ_c_ (ns)** | $\frac{D_{II}}{D_{\perp}}$ | $S_{f}^{2}$ | $S_{s}^{2}$ | **τ_s_ (ps)** | **E/N** |
| --- | --- | --- | --- | --- | --- | --- | --- |
| Model 1 | 500,600, 950 | 5.7 | 1.3 |  |  |  | 9.8 |
| Model 5 $\tau_{e}^{\max}=400 ps$ | 500,600, 950 | 6.1 | 1.2 | 0.89 | 0.91 | 393 | 8.7 |
| Model 5 $\tau_{e}^{\max}=1000 ps$ | 500,600, 950 | 6.6 | 1.3 | 0.89 | 0.83 | 954 | 3.4 |
| Model 5 $\tau_{e}^{\max}=1500 ps$ | 500,600, 950 | 7.1 | 1.2 | 0.87 | 0.77 | 1410 | 1.8 |
| Model 5 $\tau_{e}^{\max}=2000 ps$ | 500,600, 950 | 7.7 | 1.2 | 0.86 | 0.70 | 1810 | 1.3 |
| Model 5 $\tau_{e}^{\max}=3000 ps$ | 500,600, 950 | 8.3 | 1.3 | 0.84 | 0.64 | 2230 | 1.1 |
| Model 5 $\tau_{e}^{\max}=5000 ps$ | 500,600, 950 | 8.3 | 1.3 | 0.84 | 0.64 | 2230 | 1.1 |

The different parameter and model of motions are described in the text. The results have been obtained using Powell optimizations in Model-Free, the S^2^ and values are the average values of the different residues contained in N (ZF1) and C (ZF2) domains. The selected residues are those that contain at both fields neither signs of conformational exchange neither large amplitude, long time scale internal motions (characterized by low heteronuclear NOE). The relevant parameters for model 5 (that contained both a fast and slow component) are $S_{s}^{2}$ related to the slow component and . $S_{f}^{2}$ value is related to the fast motion described by the model 5, recall that with Model-Free , we cannot extract the value of the correlation time of the fast motion as it is supposed to be extremely fast. The extracted values for the slow component τ_s_ are shown.

In the calculations made with the model 5, the maximum value that the correlation time of the slow motion () can take is varied.
